# Loss of Vps54 Function Leads to Vesicle Traffic Impairment, Protein Mis-Sorting and Embryonic Lethality

**DOI:** 10.3390/ijms140610908

**Published:** 2013-05-24

**Authors:** Páll Karlsson, Aida Droce, Jakob M. Moser, Simon Cuhlmann, Carolina Ortiz Padilla, Peter Heimann, Jörg W. Bartsch, Annette Füchtbauer, Ernst-Martin Füchtbauer, Thomas Schmitt-John

**Affiliations:** 1 Molecular Biology and Genetics Department, Aarhus University, Aarhus 8000, Denmark; E-Mails: pkar@svf.au.dk (P.K.); ad@bio.aau.dk (A.D.); jmm@mb.au.dk (J.M.M.); simon.cuhlmann@gmx.de (S.C.); carolina.ortiz@post.au.dk (C.O.P.); acf@mb.au.dk (A.F.); emf@mb.au.dk (E.-M.F.); 2 Cell Biology Department, Bielefeld University, Bielefeld 33501, Germany; E-Mail: peter.heimann@uni-bielefeld.de; 3 Department of Neurosurgery, University Marburg, Marburg 35033, Germany; E-Mail: jbartsch@med.uni-marburg.de

**Keywords:** Vps54, wobbler, ALS, GARP complex, retrograde vesicle transport

## Abstract

The identification of the mutation causing the phenotype of the amyotrophic lateral sclerosis (ALS) model mouse, wobbler, has linked motor neuron degeneration with retrograde vesicle traffic. The *wobbler* mutation affects protein stability of Vps54, a ubiquitously expressed vesicle-tethering factor and leads to partial loss of Vps54 function. Moreover, the Vps54 null mutation causes embryonic lethality, which is associated with extensive membrane blebbing in the neural tube and is most likely a consequence of impaired vesicle transport. Investigation of cells derived from wobbler and Vps54 null mutant embryos demonstrates impaired retrograde transport of the Cholera-toxin B subunit to the trans-Golgi network and mis-sorting of mannose-6-phosphate receptors and cargo proteins dependent on retrograde vesicle transport. Endocytosis assays demonstrate no difference between wobbler and wild type cells, indicating that the retrograde vesicle traffic to the trans-Golgi network, but not endocytosis, is affected in Vps54 mutant cells. The results obtained on wobbler cells were extended to test the use of cultured skin fibroblasts from human ALS patients to investigate the retrograde vesicle traffic. Analysis of skin fibroblasts of ALS patients will support the investigation of the critical role of the retrograde vesicle transport in ALS pathogenesis and might yield a diagnostic prospect.

## 1. Introduction

Amyotrophic lateral sclerosis (ALS) is the most common adult-onset motor neuron disease in humans [1]. It is characterized by progressive degeneration of both upper and lower motor neurons, leading to progressive muscle weakness. The majority of ALS cases occur sporadically (sALS), and only about 10% are familial (fALS) cases with a clear inheritance [1]. Among the fALS cases, 20% are associated with dominantly inherited mutations in the superoxide dismutase 1 (SOD1) gene [2], but a few other ALS-causative genes have been identified, including ALS2/alsin [3], ALS4/senataxin [4] and ALS8/VAPB (vesicle associated membrane protein B) [5]. Even though ALS has been investigated thoroughly since first described by Charcot in 1896, very little is known about the precise pathomechanisms, a fact reflected by the unavailability of an effective therapeutic treatment.

Like the SOD1 G93A transgenic mouse, the wobbler mouse [6] is a widely used animal model for human motor neuron diseases (MND), such as ALS. We have identified the mutation responsible for the wobbler phenotype [7]. The wobbler point mutation (L967Q missense mutation) affects Vps54, a ubiquitously expressed vesicle traffic factor and thus links motor neuron degeneration to intracellular vesicle transport.

As known in yeast, Vps54 is a component of the Golgi associated retrograde protein (GARP) complex, a tethering complex involved in retrograde vesicle traffic from endosomes to the trans-Golgi network (TGN) [8]. The GARP complex is a tetrameric complex of Vps51, Vps52, Vps53 and Vps54, and the mammalian counterpart appears to have the same subunits and fulfills the same function in the retrograde vesicle traffic of mammalian cells [9–11]. Recent studies in cell culture have clearly demonstrated the important role of the GARP tethering complex for retrograde vesicle traffic [10,11]. The complete inactivation of Vps54 leads to embryonic lethality, and homozygous null mutant embryos die around day 11 of embryonic development (E11) [7]. Mutant embryos show a retarded heart development, nearly absent dorsal root ganglia, and increased signs of apoptosis, indicating the importance of the GARP complex for embryonic development. Recently, the t^w5^ allele of the murine t-complex was shown to be a Vps52 null mutation and thus has linked complete loss of Vps52 function with early embryonic lethality and gastrulation defects [12]. Since the Vps52 null mutation leads to much earlier embryonic lethality than the Vps54 null mutation one might conclude that either the Vps54 null mutation does not lead to a complete loss of GARP function or that Vps52 has a GARP independent function during gastrulation.

However, the *wobbler* point mutation is not embryonic lethal and can be considered as a hypomorphic Vps54 allele. A recent report indicates that the *wobbler* mutation causes a destabilization of Vps54 and the entire GARP complex and thus increases degradation [13]. We have demonstrated that the wobbler mutation leads to enlarged endosomal structures in degenerating motor neurons [14]. Similar structures were observed in a subset of human sporadic ALS patients [14], indicating that the destabilization of the GARP complex or similar impairments of the retrograde vesicle transport plays a role in ALS. Until now, VPS54 mutations have not been identified in human ALS and thus do not appear to be a major cause of ALS in humans [15]. However, other GARP components or other factors involved in the retrograde vesicle transport have not been screened for mutations in human ALS cases. It is therefore important to understand how the retrograde vesicle traffic is impaired in wobbler cells and what the cellular consequences are.

In order to unravel the precise pathomechanism that leads to motor neuron degeneration and inflammatory responses in wobbler mice, the contribution of other CNS cell types such as glial cells or interneurons have to be considered. In particular we have recently shown that hyperexcitability of pyramidal neurons in the wobbler motor cortex is due to a decreased number of GABA-ergic interneurons [16].

Here we investigated the effect of the Vps54 and Vps53 null mutations *in vivo* and analyzed the retrograde vesicle traffic in murine embryonic fibroblasts (MEF) derived from wild type, wobbler or Vps54 null mutant individuals. We demonstrate that wobbler mutant cells have impaired retrograde vesicle traffic, leading to mis-sorting of various proteins, and suggest that cultured skin fibroblasts (SKF) from skin biopsies could be used as a diagnostic tool to test for vesicle transport defects in ALS patients.

## 2. Results and Discussion

### 2.1. GARP Mutant Embryos

The GARP complex is a vesicle tethering complex tethering endosome-derived vesicles to the TGN. Consequently, loss of GARP function is associated with impairments in the retrograde vesicle transport [17]. Our Vps54 knockout that results in embryonic lethality around E11 [7], was thought to be due to a complete loss of GARP function. However, single, double and triple mutations of the corresponding yeast GARP core components (Vps52, 53, and 54) cause identical phenotypic effects [8]. In addition, the recent association of the Vps52 null mutation with gastrulation defects, and thus, much earlier embryonic lethality, might indicate that a complete loss of GARP function would cause gastrulation defects, and that the Vps54 knockout might not result in a complete loss of GARP function. Thus, we generated a Vps53 null mutant mouse by using an embryonic stem cell (ES) clone with a gene trap insertion in the first intron of Vps53 (RRS890; Baygenomics). Heterozygous offspring were obtained and mated, but no homozygous mice were obtained in multiple matings, suggesting embryonic lethality. Examination of embryos at different stages revealed the approximate Mendelian quarter of homozygous null mutant embryos (11 of 44) at E10.5, none of which showed an overt abnormal phenotype (not shown). However, no homozygous Vps53 null mutant embryos were found, at E11.5, only resorption sites, arguing for embryonic death between E10.5 and E11.5. A similar range was demonstrated for Vps54 knockout embryos [7], but substantially later than Vps52 null mutants [12].

From these findings, two alternative hypotheses can be drawn: One, that Vps54 or Vps53 knockouts both lead to an incomplete loss of GARP function, while the Vps52 null mutation leads to a complete loss of GARP function; or two, that the Vps54 or Vps53 knockouts both constitute GARP null mutations and the mammalian Vps52 has an additional function during gastrulation. The similarity of the time points of embryonic death of Vps54 and Vps53 mutants argues for the second possibility.

**Figure 1:**
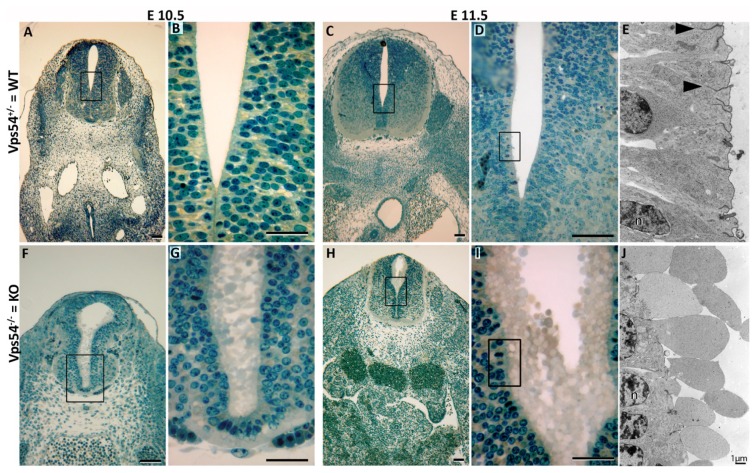
Vps54 knockout embryos show extensive membrane blebbing into the lumen of the neural tube. (**A**–**E**) cross-sections of heterozygous Vps54^+/−^ (wild type phenotype) and (**F**–**J**) Vps54^−/−^ embryos. (**A**, **B**, **F**, **G**) are E10.5 and (**C**–**E**) and (**H**–**J**) are E11.5 embryos. Semi-thin sections (**A**–**D**) and (**F**–**I**), stained with Richardson’s blue and inspected by light microscopy, (**E**) and (**J**) corresponding electron micrographs to (**E**) and (**I**). Note membrane blebs (**J**) devoid of any organelles and cytoskeletal elements. n = nucleus; arrowheads = tight junctions in apical zone of lining cells. Scale bars in **A**, **C**, **D**, **F**, **H** = 100 μm, **B**, **G**, **I** = 50 μm, **E** and **J** = 1 μm.

Examination of E10.5 Vps54 knockout embryos revealed growth retardation, retarded heart development, nearly absent dorsal root ganglia and signs for increased apoptosis as earlier stated [7]. Here we report a very specific cellular effect of the Vps54 null mutation. In the central channel of the neural tube, and to lesser extent in other epithelial tissues, we see an unusual membrane blebbing (Figure 1). The transmission electron micrographs (Figure 1) show an extensive blebbing out of membrane-enclosed nearly organelle-free cytoplasm. A weaker extracellular matrix can also be recognized. This effect is caused by the loss of Vps54, and thereby GARP function, and is probably the consequence of impairments of the retrograde vesicle transport affecting the overall membrane transport.

### 2.2. Retrograde Vesicle Traffic Is Affected in Vps54 Mutant Cells

**Figure 2:**
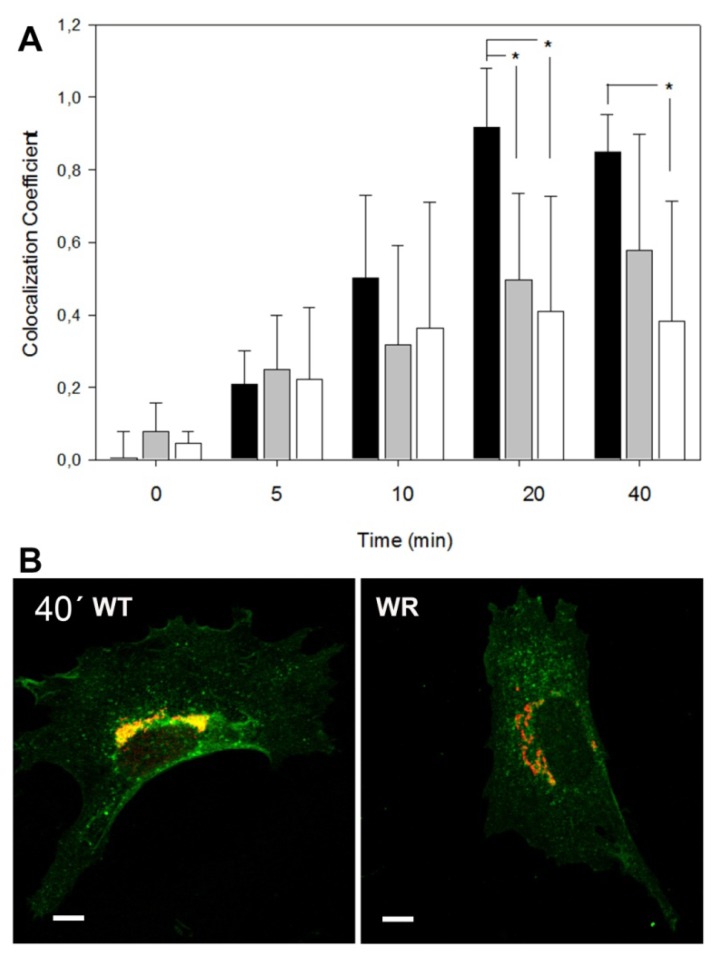
Retrograde CTB transport is affected in Vps54 mutant cells. (**A**) Quantification of Golgi-localized CTB-GFP (co-localization coefficients) after 0, 5, 10, 20 and 40 min of incubation. *Vps54*^+/+^ (WT) MEFs black bars, *Vps54^wr/wr^* (WR), grey, *Vps54*^−/−^ white bars. * indicates significant difference, *p* ≤ 0.05 *T* test (*n* = 6); (**B**) Representative immunofluorescence pictures (overlays) of WT and WR MEFs after 40 min of incubation; CTB-GFP, green, Golgi (stained with anti GM130, red), co-localization yellow, scale bars = 10 μm.

To investigate the cellular effects of partial and complete loss of GARP function, we determined up-take and retrograde transport of Cholera toxin B-subunit (CTB) in embryonic fibroblasts (MEFs) of wobbler and Vps54 knockout mutant embryos and measured the proportion of Golgi-localized CTB at different time points. Wobbler MEFs prepared on day E13.5 show a significantly slower transport of CTB to the TGN and a lower co-localization coefficient compared to WT fibroblasts (data not shown). We also included MEFs from Vps54 null mutant embryos prepared at E9.5, since Vps54 null mutant embryos die around E11 [7]. E9.5 wobbler MEFs showed a similar impairment of the retrograde CTB transport as seen in E13.5 MEFs, while Vps54 null mutant MEFs displayed an even more pronounced impairment of the retrograde CTB transport than that seen in wobbler MEFs (Figure 2).

The CTB assay showed a slower retrograde CTB transport to the TGN in Vps54 mutant MEFs, which is in agreement with the results from Perez-Victoria *et al.* [10], who showed similar effects on Shiga toxin transport in Vps52 depleted Hela cells. However, this might also reflect impaired endocytosis.

### 2.3. Endocytosis Is Not Affected in Vps54 Mutant Cells

**Figure 3:**
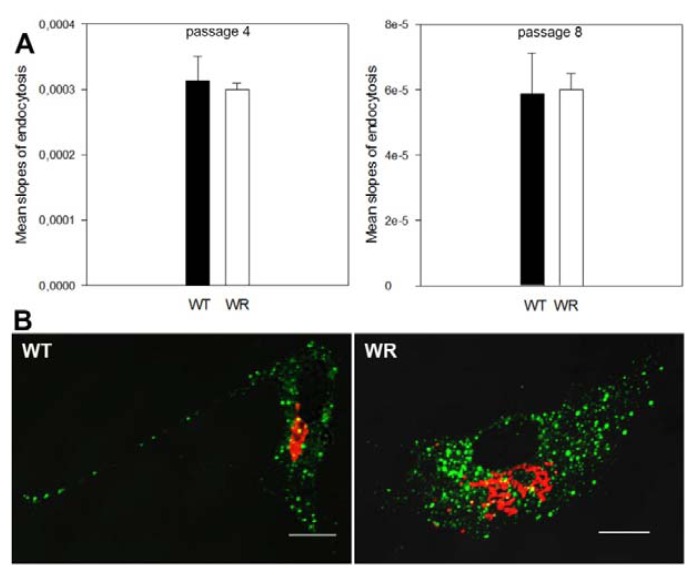
Endocytosis is not affected in Vps54 mutant cells. (**A**) HRP fluid phase endocytosis assay; quantification of the activity of internalized HRP after 40 min incubation of wild type (WT; black) and wobbler (WR; white) MEFs at passage 4 and 8 respectively. No significant difference could be detected; (**B**) Representative immunofluorescence pictures of wild type and wobbler MEFs staining for co-internalized ci-M6PR anti ci-M6PR antibody (green), Golgi staining (anti GM130; red); scale bars = 10 μm. No obvious difference between WT and WR MEFs could be detected.

The CTB assay reveals a slower CTB transport to the TGN in Vps54 mutant MEFs, but this might also reflect impaired endocytosis. To investigate if endocytosis was affected in wobbler MEFs, we performed a fluid-phase endocytosis assay (Figure 3a). No difference in endocytosis was seen between wobbler and wild type E13.5 MEFs. Thus, we conclude that the effect of mutant Vps54 is restricted to the retrograde vesicle traffic from endosomes to the TGN and does not affect endocytosis. In addition, the endocytosis of Mannose-6-phosphate receptors (M6PR) was tested in wobbler and wild type MEFs by visualizing the co-internalization of a specific antibody with surface located cation-independent M6PR. No difference was detected between wobbler and wild type cells (Figure 3b), additionally indicating that the endocytosis is not affected by the Vps54 mutation.

Our results revealed statistically significant differences in retrograde CTB transport between wild type and Vps54 mutant MEFs, which is not due to a defect of endocytosis in Vps54 mutant cells. Thus, we conclude that the differences in CTB localization are due to intracellular transport defects.

### 2.4. Protein Mis-Sorting in Vps54 Mutant Cells

**Figure 4:**
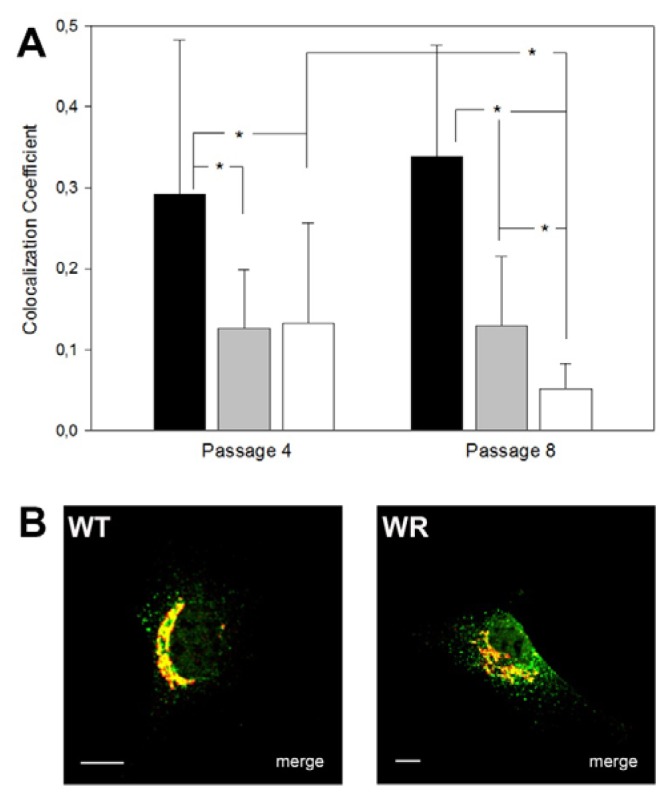
M6PRs are mislocalized in Vps54 mutant cells. (**A**) Quantification of Golgi-localized ciM6PRs (co-localization coefficients) of wild type (black), wobbler (grey) and Vps54 KO (white) MEFs from passage 4 and 8. Differences, significant using ANOVA (*p* ≤ 0.01) are indicated (*), *n* = 10; (**B**) Immunofluorescence overlay pictures of representative WT and WR MEFs (P4); immunostaining for ciM6PR, green, Golgi, red; scale bars = 10 μm.

We have recently shown that APP protein accumulates in abnormally enlarged endosomal structures in degenerating motor neurons in wobbler mice and also in a subset of human sporadic ALS cases [14]. In Vps54 knockdown cells mis-sorting of lysosomal enzymes and the sorting receptors M6PR was reported [10], indicating that full or partial loss of GARP function leads to mis-sorting of proteins and probably to a depletion of Golgi resident sorting receptors. Thus, we investigated the sub-cellular distribution of the cation-independent mannose-6-phosphate receptor (ciM6PR) in MEFs from E13.5 and E9.5 wobbler and Vps54 null-mutants by immunostaining and measured the proportion of Golgi-localized ciM6PR. In addition, since motor neuron degeneration in wobbler mice is progressive, we included embryonic fibroblasts from passage 4 and 8 to test if misdistribution of ciM6PR aggravates over time. In E13.5 wobbler MEFs a significantly lower proportion of Golgi-localized ciM6PR was observed (mean co-localization coefficient was 0.2 and 0.4 in E13.5 wobbler and WT MEFs respectively, *N* = 30, *p* < 0.00001). Similar results were obtained for E9.5 wobbler and Vps54 null mutant MEFs (Figure 4). When comparing distribution of ciM6PR in E9.5 MEFs of passage 4 and 8 no difference was detected for P4 and P8 wobbler MEFs. However, E9.5 Vps54 null mutant MEFs showed a significant reduction in the amount of Golgi-localized ciM6PR at passage 8 as compared to passage 4, indicating a cumulative effect of M6PR mis-sorting (Figure 4).

These results fit well with the earlier reported mis-sorting of ciM6PRs in Vps52 depleted Hela cells [10]. In order to analyze the distribution of the cation-dependent M6PR (cdM6PR), we generated a cdM6PR-EGFP-fusion construct. E13.5 wobbler or wild type MEFs were transfected with the cdM6PR-EGFP-fusion construct. Wobbler MEFs displayed a similar misdistribution of the cdM6PR, as demonstrated for the ciM6PR (co-localization coefficients of WT cells = 0.3 and WR = 0.1, *N* = 30, *p* < 0.001).

These results indicate that mutation of Vps54 results in an impaired retrograde vesicle transport and thereby inhibits the transport of ciM6PRs and cdM6PRs back to the TGN, which results in a more dispersed distribution in endosomes and depletion of Golgi-localized M6PRs.

**Figure 5:**
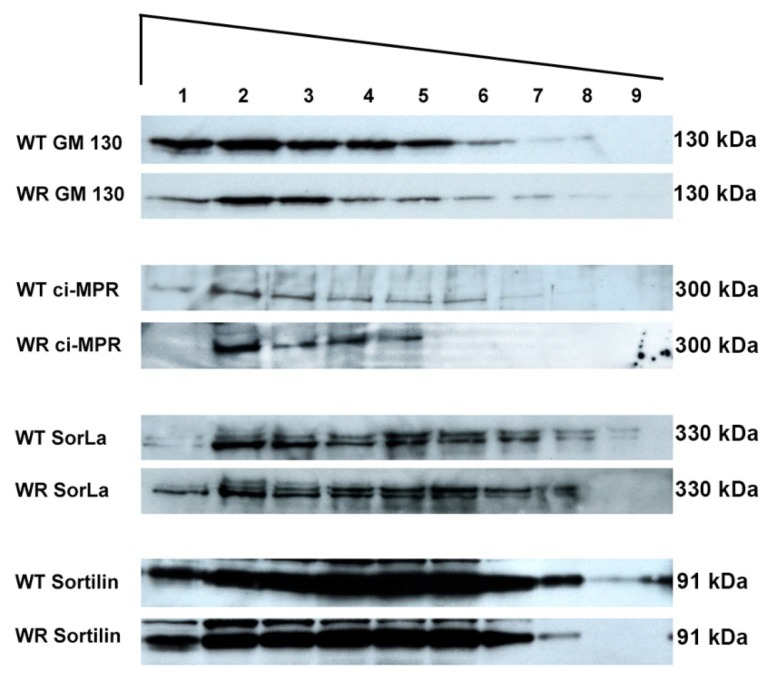
Western blot analysis of size fractions of membrane compartments of WT and WR brains. Membrane compartments from wild type (WT) and wobbler (WR) brain extracts were separated according to size into 20 fractions. Every second fraction was loaded onto a SDS-PAGE (Lane 1 = F2, 2 = F4, 3 = F4, 4 = F8, 5 = F10, 6 = F12, 7 = F14, 8 = F16, 9 = F18) the largest compartments are fractions F1 and F2 whilst F18 to F20 represent the smallest. Immunoblots for GM130 (Golgi), ciM6PR, SorLa and Sortilin are shown and differences in the pattern of the various proteins between WT and WR indicates misdistribution. For M6PR, SorLa and Sortilin sorting receptors differences are detectable.

To obtain a broader overview of mis-sorted proteins, we used Western blot analyses of size fractionated vesicular or membrane compartments (Figure 5). Using wobbler and wild type brain tissue such an assay should also confirm the mis-sorting of M6PRs *in vivo*. The different size fractions were loaded on SDS PAGE and analyzed by immunoblotting and the amounts of the various proteins in size fractions was monitored and quantified (Figures 5 and S1).

Sorting receptors like M6PRs, SorLa or Sortillin appear to be differentially distributed between wobbler and wild type fractions, while no obvious misdistribution was found for Golgi-marker proteins (GM108) and GARP components (Vps53; Figure S1). However, quantification of GARP component Vps53 revealed a reduced amount of GARP proteins in wobbler tissue, arguing for decreased GARP stability in wobbler brains (Figure S1), but much less pronounced as recently demonstrated in Vps52 knockdown experiments [13].

**Figure 6:**
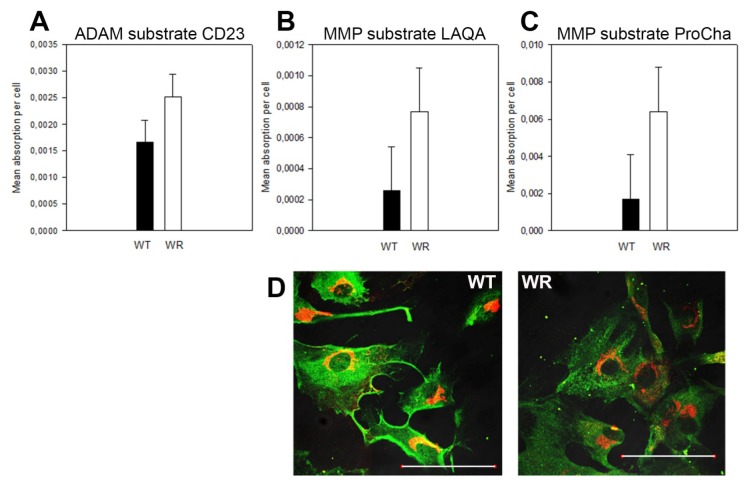
Surface located proteins are affected in Vps54 mutant MEFs. (**A**) CD23 substrate detects activities of ADAM8/MMP2 in MEF supernatant. A slightly higher ADAM activity is found in WR MEF supernatant, statistically significant, *p* = 0.042, two-tailed *T*-test; (**B**) MMP substrate LAQA revealed increased MMP activity in WR MEF supernatant, however, not significant (*p* = 0.0586); (**C**) MMP substrate ProCha shows significantly higher MMP activity in WR MEF supernatant (*p* = 0.0137); (**D**) Immunostaining for endogenous APP (green; Golgi, red) revealed a higher proportion of surface located APP in WT MEFs as compared to WR; scale bars = 100 μm.

Mislocalization of cargo proteins like APP have previously been shown in degenerating motor neurons of wobbler mice [14]. Thus, the APP localization in cultured wobbler and wild type MEFs was analyzed and we observed a decreased proportion of surface localized APP (Figure 6d), but APP was not found in enlarged endosomal structures as shown previously in wobbler spinal cord sections [14].

If transport of surface proteins like APP and sorting receptors appear to be affected by the Vps54 mutation one can assume that other surface localized proteins might be mis-sorted as well. To test this, activities of surface localized proteases like ADAMs and MMPs were tested, since elevated activity levels of MT-MMP1 and ADAM8 have previously been reported in the wobbler CNS [18,19]. These activities most likely reflect neuroinflammatory reactions, but could also be due to a disturbed removal of activated MMPs/ADAMs from the surface. Since neuroinflammatory processes can be excluded in MEFs, it is interesting to note that we found significantly increased activated MMP/ADAM activities in the supernatant of wobbler MEFs as compared to wild type MEFs (Figure 6a–c). This difference in protease activities is likely to reflect a defect in ADAM/MMP turnover/transport rather than an inflammatory process.

Impairment of retrograde vesicle transport appears to affect directly the sorting and distribution of proteins dependent on this transport route. Perez-Victoria *et al.* [10] could show defects in sorting of cathepsin D to lysosomes in GARP-depleted cells and our results indicate, that sorting receptors (M6PR, Sortilin, SorLa) and cargo proteins like APP or surface located proteins (MMPs) are affected, while Golgi-resident proteins (GM130, GARP components) are not mis-sorted.

### 2.5. Use of Skin Fibroblasts for the Analysis of Vesicle Traffic Defects

**Figure 7:**
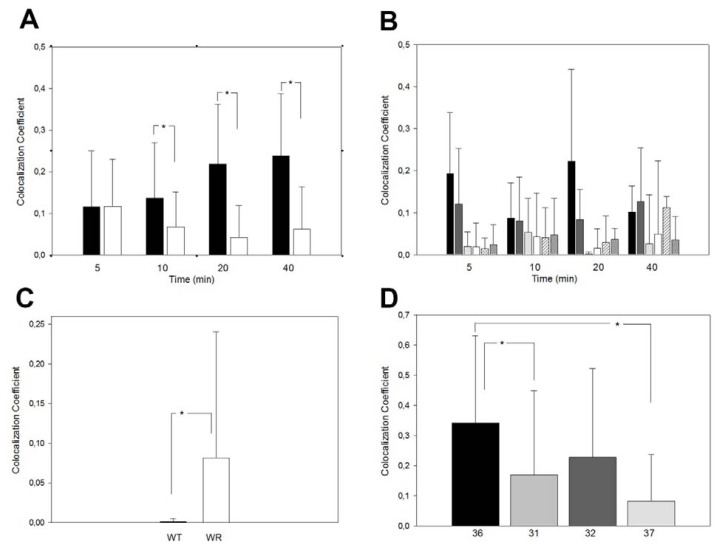
CTB- and M6PR tests in murine and human SKFs. (**A**) CTB transport test with wild type (black) and wobbler (white) SKFs, showing Golgi-localized CTB (co-localization coefficients) after 5, 10, 20 and 40 min incubation, * indicates statistically significant differences (*p* ≤ 0.05, *n* = 20); (**B**) CTB transport tests with human healthy controls and SOD1 mutant ALS patient SKFs. Bars from left to right: controls 36, 35, ALS patients 31, 32, 37 and 38. Even though a higher individual variation can be seen, at least for 5, 20 and 40 min values significant differences between controls and patients (*p* ≤ 0.05, *n* = 18) were found; (**C**) Proportion of Golgi-localized ciM6PR in wild type and wobbler SKFs (ANOVA: *p* = 0.045, *n* = 17); and (**D**) in human SKFs from control (36) and ALS patients 31, 32 and 37; * indicates *p* < 0.05 (ANOVA), *n* = 15.

Some sALS patients show enlarged endosomal structures similar to the structures found in wobbler motor neurons [14]. These enlarged structures might be caused by impairments of the retrograde vesicle traffic and thus, suggest that the retrograde vesicle transport might be affected in at least a subset of human ALS cases. To address this, we investigated the diagnostic/prognostic use of the above-mentioned assays for the investigation of vesicle traffic defects in cultured SKFs from skin biopsies. We established skin fibroblast cultures from wobbler and wild type mice and confirmed that the effects on retrograde transport and ciM6PR misdistribution seen in MEFs was also seen in SKFs (Figure 7a,c). Our results demonstrate that cultured SKFs can be used to screen for wobbler-like impairments of the retrograde vesicle traffic. Thus, we performed an initial small-scale screening test of human SKF cultures derived from skin biopsies of ALS patients and two healthy relatives as controls. We obtained exclusively SKFs from SOD1-mutant fALS cases, where we did not expect to find pronounced vesicle traffic defects. Skin fibroblasts derived from SOD1 fALS patients also displayed impairment in the retrograde vesicle traffic as well as a misdistribution of the ciM6PR similar to that seen in Vps54 mutant MEFs and SKFs (Figure 7b,d). However, the individual variations of these were much more pronounced among human patients due to genetic variance, as compared to inbred mice.

We demonstrated that patient skin fibroblasts can be used for the functional investigation of cellular vesicle traffic. Skin fibroblasts have two advantages; they can be easily obtained from skin biopsies and, due to their flattened morphology, they are nearly two-dimensional, which makes microscopic analysis of intracellular transport processes more reliable than a three-dimensional analysis. Thus, human ALS patients can be screened for “wobbler-like” vesicle traffic defects, which is of high diagnostic and prognostic value.

All four fALS patients investigated so far seem to bear defects in the retrograde vesicle traffic and, as judged by confocal microscopy, there is no noticeable difference in the CTB uptake itself (data not shown). The results presented here lead to the conclusion that at least for the patients investigated in this study, ALS patients with SOD1 mutations have vesicle traffic defects, which can be detected using skin fibroblasts. This is not unexpected, since SOD1 mutations previously have been associated with vesicle traffic defects [20], axonal transport defects and an interaction of mutant SOD1 with the Dynein motor complex [21] have been reported. Furthermore, gene expression analysis of Kirby *et al.* [22] also suggests a link between SOD1 mutation and vesicle traffic. Thus, we conclude that our observations on human SOD1 mutant skin fibroblasts merely reflect an effect of SOD1 mutation on the retrograde vesicle traffic, which should be confirmed by analyzing a larger cohort of ALS patients.

## 3. Experimental Section

### 3.1. Mice and Diagnostics

Breeding and PCR diagnosis of C57BL/6J-Vps54*^wr^*, C57BL/6J-*Vps54*^β^*^-geo^* and C57Bl/6J stock mice has been described earlier [7]. Gene trap ES cell line RRS890 with a β-Geo insertion in intron 1 of Vps53 was obtained from Baygenomics and used to generate heterozygous *Vps53*^β^*^-geo^* mice, which were backcrossed over 10 generations to C57BL/6J, thus referred to as C57BL/6J-*Vps53*^β^*^-geo^*. Mice were diagnosed for presence of the β-geo allele. Heterozygous *Vps53*^+/β^*^-geo^* mice were mated, the appearance of a vaginal plug was considered day E0.5 of embryonic development. At E10.5 and E11.5 embryos were isolated in ice-cold 1 × PBS and photographically documented. Embryos were genotyped by allele quantification using qPCR to distinguish heterozygous from homozygous embryos as described in [23]. Primers were forward: GACCGCTGGGATCTGCCATTGTCAGACATG and reverse: CCATGTGCCTTCTTCCGCGTGCAGCAGATG. Animal breeding and handling was done in accordance with the Danish law and with a permit by the local authorities.

### 3.2. EM and Light Microscopy

Preparation of mice, semi-thin sectioning, Richardson’s blue staining and electron microscopy were performed as previously described [24,25].

### 3.3. Culture of MEFs and SKFs

MEFs were derived from either E13.5 or E9.5 day embryos. Embryonic fibroblasts were prepared according to Nagy *et al.* [26].

For preparing murine SKFs, abdominal hairs were shaved off the mice and pieces of the outer layer of the skin cut off and transferred to a Petri dish containing Hanks Balanced Salt Solution (HBSS, Gibco, Life Technologies, Carlsbad, CA, USA) supplemented with antibiotics. Approximately 1 mm^3^ skin pieces were seeded out on a gelatin coated 35 mm cell culture plate and DMEM medium was added after short air-drying. Cultures were incubated at 37 °C and 5% CO_2_. Medium was changed every 5–7 days until around 50% confluence was achieved. The cells were then passaged and used for tests or storage.

Mouse SKFs (WR and WT) were grown in Dulbecco’s Modified Eagle Medium (DMEM; with l-Glutamine, 4500 mg/L Glucose, without Sodium Pyruvate; Gibco 41965), with 20% fetal calf serum (FCS) and PenStrep (Gibco, Life Technologies, Carlsbad, CA, USA). MEFs were grown in same medium except that FCS volume was lowered to 10%.

### 3.4. Human SKF Lines

Human cell lines derived from skin biopsies of fALS patients and unaffected relatives were obtained from Peter M. Andersen, Umeå University, Umeå, Sweden; ALS 31 (SOD1 G93S), ALS 32 (SOD1 H46R), ALS 35 (healthy control), ALS 36 (healthy control), ALS 37 (SOD1 G127x), and ALS 38 (SOD1 G127x). Human skin fibroblasts were grown in a RPMI 1640 (Gibco 52400, Life Technologies, Carlsbad, CA, USA) containing 10% fetal calf serum and supplemented with penicillin. All cell types were grown at 37 °C with 5% CO_2_ and medium was changed every 2nd or 3rd day.

### 3.5. Choleratoxin Transport Assay

MEFs and SKFs were grown on gelatin-coated coverslips in 6-well plates to yield 3.5 × 10^4^ cells per well. Cells were washed with CO_2_-independent medium (Optimen containing 2 mM l-Glutamine, Gibco, Life Technologies, Carlsbad, CA, USA) and the medium replaced with CO_2_-independent medium containing 1 μg/mL FITC-labelled ß-subunit of Cholera-toxin (CTB, Sigma, St. Louis, MO, USA) and kept on ice for 30 min. Cells were incubated for 0, 5, 10, 20 or 40 min at 37 °C, washed three times with 1 × PBS and fixed with 4% paraformaldehyde in PBS for 20 min. Cells were permeabilized with 0.2% Triton X-100 in PBS. Golgi-apparatus was visualized by immunostaining with anti GM130 antibody (1:800, BD Biosciences, Franklin Lakes NJ, USA) and Cy3-anti mouse (1:400, Dianova, Hamburg, Germany). Cells were inspected by confocal laser microscopy using a LSM510 Meta (Carl Zeiss, Jena, Germany). Co-localization coefficients were calculated for single cells using ZEN software (Carl Zeiss, Jena, Germany) and Student’s *T*-test was applied for statistical analysis. Co-localization coefficients reflect the proportion of Golgi-localized CTB.

### 3.6. Mannose-6-Phosphate Receptor Assay

MEFs or SKFs were grown as described above on coverslips in 6-well plates. Cells were fixed and permeabilized as described above and co-immunostained for cation-independent mannose-6-phosphate receptor (ci-M6PR) using a polyclonal rabbit antibody (1:400, obtained from A. Nykjær, Aarhus University) and Golgi was stained with a mouse anti GM130 antibody (1:800, BD Biosciences, Franklin Lakes, NJ, USA). Secondary antibodies were FITC-anti-rabbit (1:400, Dianova, Hamburg, Germany) and Cy3-anti-mouse (1:400, Dianova, Hamburg, Germany). Cells were treated as described above, inspected by confocal microscopy and co-localisation coefficients were measured. Co-localization coefficients reflect the proportion of Golgi-localized M6PR. A fusion construct was generated to detect cdM6PR an EGFP. Mouse cation-dependent mannose-6-phosphate receptor (cd-M6PR) was amplified with primers (AAGCTTTCCCGTGACACAATGTTCCC) and GGGCCCTTCATTGGTAGCAGATGATCA), subcloned in pCR2.1-Topo (Invitrogen, Life technologies, Carlsbad, CA, USA) and transferred in pMC EGFPa [27] using ApaI and HindIII restriction sites. The resulting expression plasmid was verified by sequencing and transfected in MEFs (3 μg DNA for 1.5 × 10^5^ cells) using Lipofectamine Plus reagent (Invitrogen, Life technologies, Carlsbad, CA, USA). The expressed cd-M6PR-EGFP fusion protein was detected by EGFP fluorescence and combined with GM130 Golgi-immunostaining as described above.

### 3.7. HRP Fluid Phase Endocytosis Assay

Fluid phase endocytosis assay was performed with WT and WR MEFs according to Tancini *et al.* [28]. Wild type and wobbler MEFs were incubated with horseradish peroxidase (HRP) in DMEM and resuspended in 40 mM potassium phosphate buffer containing 0.5% (*v*/*v*) Triton X-100, pH 6.8 on ice. The cell lysates were centrifuged and the supernatant used to determine HRP activity with 2,2′-Azino-bis(3-ethylbenzothiazoline)-6-sulfonic acid (ABTS) as a substrate. 4 μL of the diluted extract was added to 1 mL of 0.7 mM ABTS in 100 mM potassium phosphate buffer, pH 5.0 and 30 μL of 0.3% H_2_O_2_ added to the solution. The cells absorbance at 405 nanometers was recorded for 4 min ΔA_405nm_/min and their absorbance calculated using the maximum linear rate. The same procedure was performed with untreated cells as negative control.

### 3.8. Antibody Co-Internalization

WT and WR MEFs at passage 4 and 8 were seeded on gelatin coated chamber slides (Lab-Tec) and grown as described above to 60% confluency. Cells were washed with 1 × CO_2_ independent medium (CO_2_-IM, Gibco, #18045, Life technologies, Carlsbad, CA, USA) with 2 mM l-Glutamine and blocked with 1% bovine serum albumin (BSA), 10% NCS in 1 × CO_2_-IM) for 1 h at RT. Antibody (a rabbit polyclonal anti ci-M6PR; 1:1600 in CO_2_-IM) was added to 4 of the wells for each cell type, while in 4 wells only medium was added as control. Cells were incubated on ice for 30 min and washed 3 times with CO_2_-IP. Pre-warmed medium was added to each chamber at time point 0 and placed in the incubator at 37 °C. After 0 and 40 min the internalization was stopped by quick washing the cells 3 times with 1 × PBS and fixation with 4% paraformaldehyde for 20 min at RT. Fixed cells were permeabilized with 0.2% Triton X-100 1 × PBS for 10 min at RT, washing stained with monoclonal mouse anti-GM130 antibody (1:800 in 1 × PBS; BD Biosciences Franklin Lakes, NJ, USA) for 45 min followed by staining with secondary antibodies, goat anti-mouse-Cy3 (1:400; Dianova, Hamburg, Germany) and anti rabbit-Alexa488 (1:400, Dianova, Hamburg, Germany) and embedded in DAPI mounting medium (Invitrogen, Life Technologies, Carlsbad, CA, USA).

### 3.9. Cell Fractionation and Western Blotting

Size fractionation of membrane compartments were performed as described in [29] with some modifications. Three brains from 2.5 month old wild type and wobbler mice were homogenized in a Thomas® Teflon Pestle Tissue homogenizer containing 10 mL solution A (8.5% sucrose dissolved in H_2_O, 1 mM EDTA, 20 mM Hepes, PH 7.4) and 400 μL protease inhibitor mix Complete (Roche, Basel, Switzerland). Brain membrane/protein suspension was centrifuged (10 min, 1000 × *g*) and the supernatant was centrifuged an additional two times (10 min, 3000 × *g* and 15 min 20,000 × *g*, T70.1 rotor). The pellet was subsequently resuspended in 576 μL solution A together with 24 μL protease mix Complete and disrupted by passages through a 18-gauge needle followed by passages through 27-gauge needle. One hundred microlitres of this sample was used as control later. 500 μL of homogenate were separated by centrifugation for 18 h at 107,000 × *g* in a SW41 rotor (XL-A Beckman, Pasadena, CA, USA) on a continuous 0.8 M to 1.6 M sucrose gradient. The samples were then fractionated in 24 fractions and every second fractionation was loaded on a SDS-PAGE electrophoresis gel and transferred to a Western blot membrane.

The following antibodies used for immunodetection: GM130 (mouse, BD bioscience, Franklin Lake, NJ, USA, #610822), CI-MPR (rabbit, Anders Nykjær), SorLA (Rabbit, Anders Nykjær), Sortillin (Rabbit, Anders Nykjær).

### 3.10. APP Immunostaining

Fifteen thousand wobbler or wild type MEFs were seeded out on gelatin coated chamber slides (Lab-Tec). At 60% confluency the cells were washed, fixed with 4% paraformaldehyde in PBS for 20 min at RT and permeabilized with 0.2% Triton X-100 in PBS for 10 min at RT. Primary antibodies against APP-*N*-terminus (rabbit, 1:200, Sigma, A8717,) and GM130 (mouse, 1:400, BD Bioscience) diluted in PBS were incubated for 1 h. Chamber slides were washed and incubated with secondary antibodies, anti-mouse Cy3 (1:400, Dianova, Hamburg, Germany) and anti-rabbit Alexa488 (1:400, Dianova, Hamburg, Germany) for 1 h at RT. Slides were washed and cover slips were mounted with DAPI containing mounting medium (Invitrogen, Life Technologies, Carlsbad, CA, USA, S36938).

### 3.11. Quantification of Active Membrane-Bound and Secreted Metalloproteases

WT and WR MEFs at passage 4 were seeded out on 6 well plates and incubated at 37 °C until they reached confluence. Growth medium was replaced by DMEM without serum (1 mL/well). After 12 h incubation, culture media were collected, concentrated by centrifugation (VivaSpin columns with 10 kD molecular weight cut-off) and subjected to MMP activity assays using the following fluorescent substrates [30]: CD23 substrate, Dabcyl-His-Gly-Asp-Gln-Met-Ala-Gln-Lys- Ser-Lys(FAM)-NH_2_; TNF substrate, Dabcyl-Leu-Ala-Gln-Ala-Homophe-Arg-Ser-Lys(FAM)-NH_2_; MMP substrate, Dabcyl-Pro-Cha-Gly-Cys(Me)-His-Ala-Lys(FAM)-NH_2_. All substrates were diluted from a 10 mM stock in DMSO to 10 μm in assay buffer containing 50 mM Tris, pH 7.5, 150 mM NaCl, 2 mM CaCl_2_, 5 μM ZnSO4, and 0.01% Brij-35, and 10 mM CaCl_2_. Concentrated supernatants were incubated with fluorescent substrates in assay buffer and fluorescence was determined in 5 min intervals for 1 h with excitation and emission wavelengths of 485 and 530 nm, respectively. CD23 was cleaved preferentially by ADAM8 and MMP-2, ProCha indicates mainly MMP-9 activity, and LAQA detects preferentially ADAM17 (TACE), ADAM12, and most MMPs. To verify that the measured activities were derived from MMPs, samples were run in the presence of 0.1 μM BB-94, a broad-range MMP/ADAM inhibitor, which resulted in no detectable activities in all samples.

## 4. Conclusions

Complete loss of GARP function leads to embryonic lethality in mice around E11 as demonstrated for Vps54 and Vps53 null mutants or earlier during gastrulation as shown in Vps52 null mutants. Vps54 deficiency causes membrane blebbing from neural tube cells, which was first reported here. Embryonic lethality and membrane blebbing might reflect impairments of vesicle trafficking. Using MEFs from Vps54 null mutants, wobbler mutants and Wild-type mice we demonstrated that either complete or partial loss of Vps54 causes impairments of retrograde vesicle traffic and results in protein misdistribution. The CTB transport assay and the M6PR distribution test was used to investigate vesicle traffic defects in cultured skin fibroblasts and thus, affords the screening of human ALS patients for “wobbler-like” vesicle traffic defects. Taken together with ALS genes such as VAPB and Alsin, which have linked ALS to vesicle traffic, our present results provide further evidence that vesicle traffic plays a major role in ALS pathology. Analysis of vesicle traffic defects in cultured skin fibroblasts from skin biopsies may serve as a differential diagnostic tool in the future and forms a platform for the development and testing of new therapeutic strategies.

## Supplementary Information
