# Supplementary material for: Loss of Vps54 Function Leads to Vesicle Traffic Impairment, Protein Mis-Sorting and Embryonic Lethality
Source: Int J Mol Sci. 2013 May 24;14(6):10908–25. doi: 10.3390/ijms140610908 (PMC3709709; doi:10.3390/ijms140610908)
Supplement: Supplementary file 1 [file ijms-14-10908-s001.pdf]

Supplementary Information

Figure S1. Vps53 is not mis-distributed but less abundant in *Vps54*$^{wr/wr}$ brains. Vps53 isoforms in size fractionated membrane compartments form wilt type (WT) wobbler (WR) brain extracts. Every second fraction, fraction 2 to 20 was separated on SDS-PAGE, Western blotted and immunostained for Vps53 (rabbit anti Vps53, ABIN). The each band was quantified by densitometry and WT (green) and WR (purple) Vps53 isoforms were plotted separately.

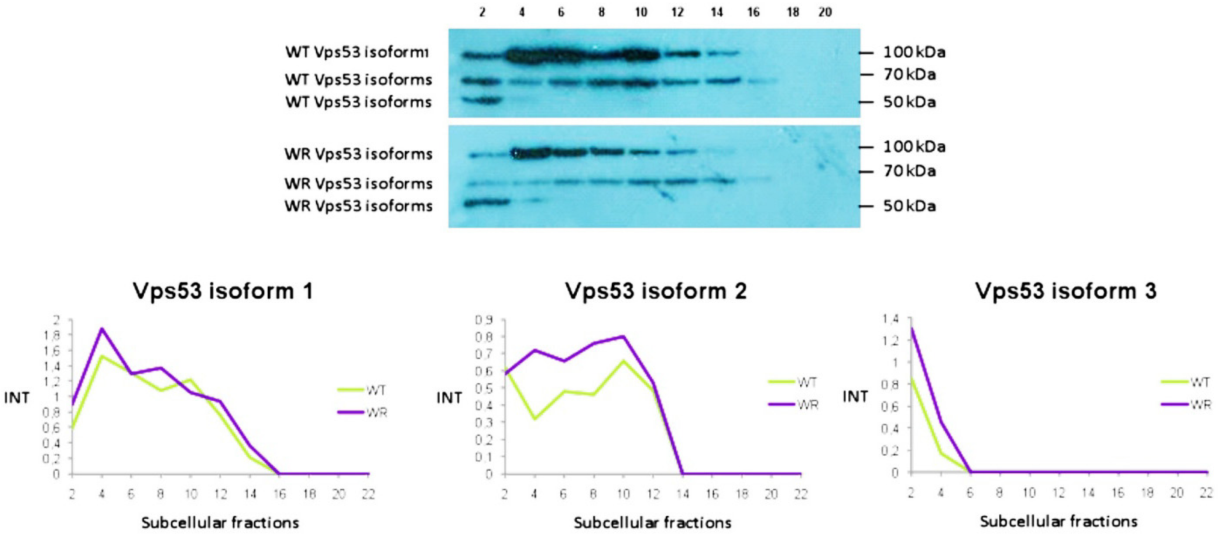

© 2013 by the authors; licensee MDPI, Basel, Switzerland. This article is an open access article distributed under the terms and conditions of the Creative Commons Attribution license (http://creativecommons.org/licenses/by/3.0/).
